# Burst fracture treatment caudal to long posterior spinal fusion for adolescent idiopathic scoliosis utilizing temporary lumbo-pelvic fixation with restoration of lumbar mobility after instrumentation removal

**DOI:** 10.1016/j.xnsj.2023.100307

**Published:** 2023-12-30

**Authors:** Mohammad Daher, Shelby Cronkhite, Mariah Balmaceno-Criss, Abel De Varona-Cocero, Bassel G. Diebo, Alan H. Daniels

**Affiliations:** Department of Orthopaedic Surgery, Brown University, Providence, Rhode Island

**Keywords:** Scoliosis, Adolescent idiopathic scoliosis, Mobility, Burst fracture

## Abstract

**Background:**

Thoracolumbar burst fractures are common traumatic spinal fractures. The goals of treatment include stabilization, prevention of neurologic compromise or deformity, and preservation of mobility. The aim of this case report is to describe the occurrence and treatment of an L4 burst fracture caudal to long posterior fusion for adolescent idiopathic scoliosis (AIS).

**Case report:**

A 15-year-old girl patient underwent posterior spinal fusion from T3–L3. The patient tolerated the procedure well and there were no complications. Seven years postoperatively, the patient reported to the emergency department with lumbar pain after fall from height. A burst fracture at L4 was diagnosed and temporary posterior instrumentation to the pelvis was performed. One-year postinjury, the hardware was removed with fixation replaced only into the fractured segment. Flexion/extension radiographs revealed restored motion.

**Conclusions:**

Treatment of fractures adjacent to fusion constructs may be challenging. This case demonstrates that avoiding fusion may lead to satisfactory outcomes and restoration of mobility after instrumentation removal.

## Introduction

Thoracolumbar burst fractures are a common type of traumatic spinal fracture, accounting for more than 2/3 of thoracolumbar fractures [1]. The goal of treatment for thoracolumbar burst fractures includes stabilization with or without decompression to deter progressive deformity and neurologic compromise [2]. However, debate still exists over the optimal treatment for this kind of fracture and how to preserve the most mobility [3]. Instrumentation plays a role in restoring immediate stability and correcting the deformity. Past research has demonstrated that mobility in the affected segment is more likely to be preserved in patients who do not undergo fusion for thoracolumbar burst fracture [4]. Several studies demonstrated that posterior fixation without fusion may reduce operation time, blood loss, and help avoid donor site complications [5], [6], [7]. However, in the long term, solid fusion may be required to prevent instrumentation failure and no data exists regarding fractures adjacent to long segment fusion [8]. In fact, Chou et al.[9] reported a higher rate of revision surgery in patient who did not undergo fusion. Nevertheless, a meta-analysis by Diniz et al. [4] reported no statistically significant difference in the rate of reoperation. Given the concern for both achieving stability and maintaining mobility, the question of whether to fuse the spine in posterior instrumentation of burst fractures is controversial.

Traumatic vertebral fracture caudal to long segment posterior fusion and instrumentation for AIS is a rare occurrence and a limited number of cases have been described in the literature [10], [11], [12], [13]. In these cases, the question of whether to treat the fracture with fusion becomes even more complex; given the balance between protecting the previous construct and preserving mobility in patients that may have already lost spinal motion in previous operations. Here we present the case of a patient treated with posterior spinal fusion for AIS who sustained a fracture at L4 following T3–L3 posterior instrumented fusion for AIS.

## Case presentation

**Fig. 1:**
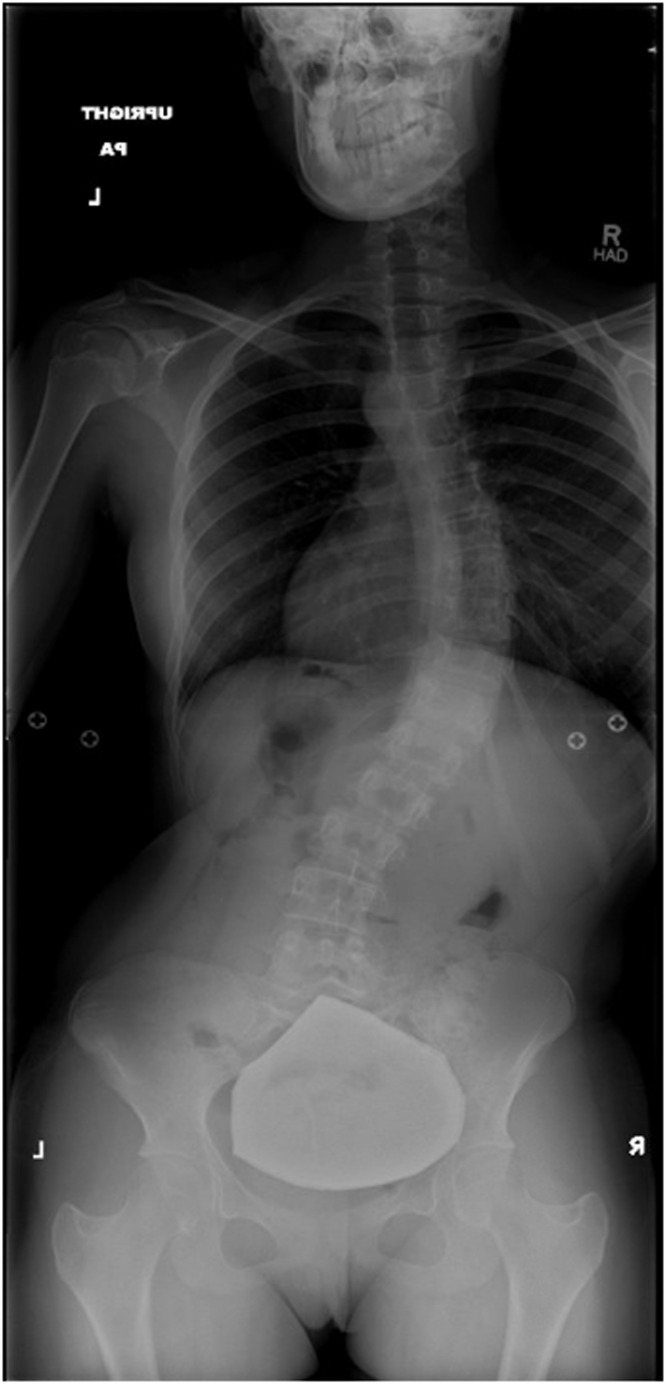
Anteroposterior plain radiograph film showing a 49° dextroscoliosis of the thoracolumbar spine centered at T9/T10 and a 30° levoscoliosis of the lower lumbar spine centered at L4.

**Fig. 2:**
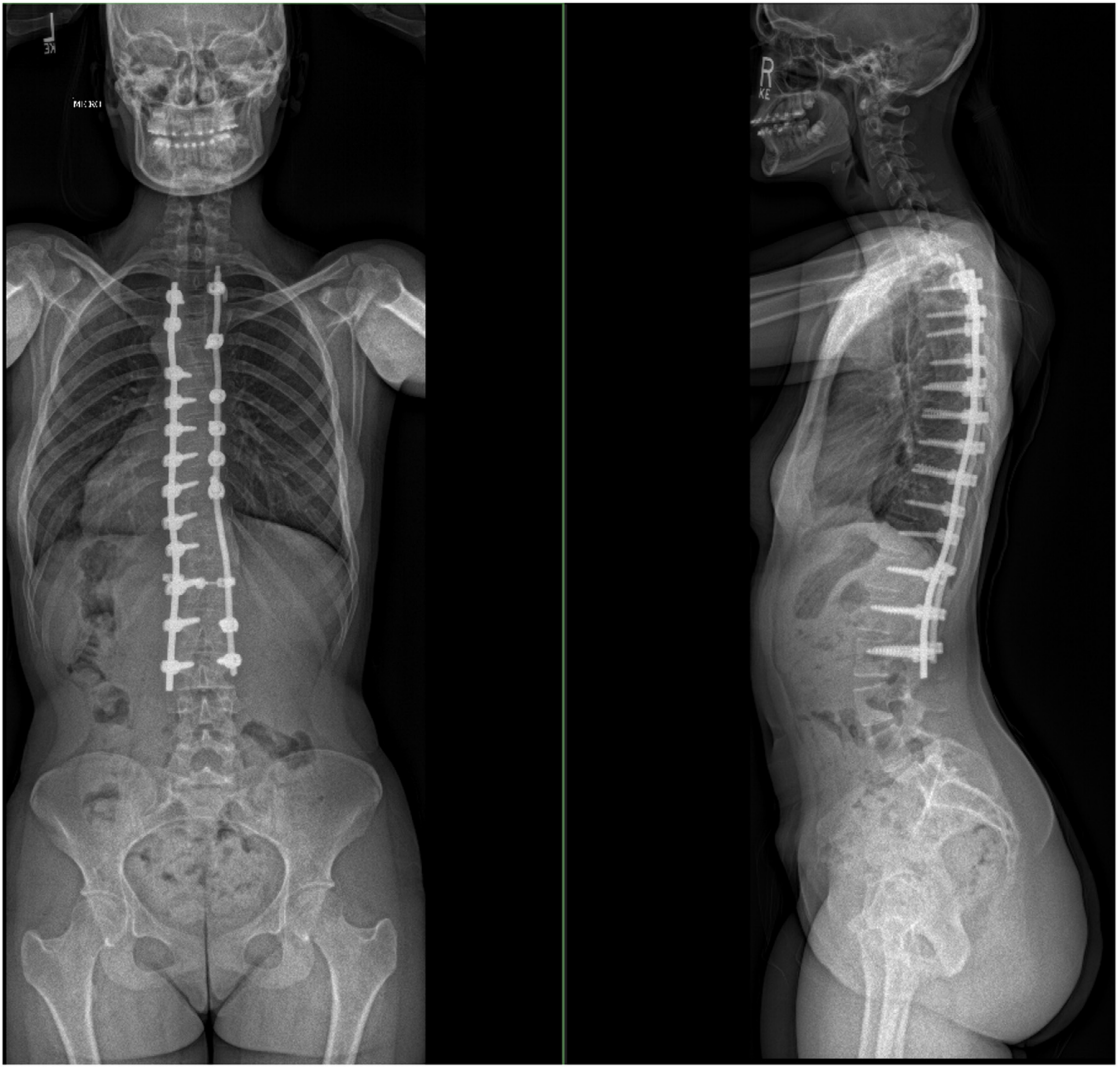
Anteroposterior and lateral plain radiograph showing correction of scoliosis with a posterior instrumentation from T3 t– L3.

A 15-year-old girl presented for evaluation of AIS in 2015. The patient complained of constant worsening right back pain. 36-inch radiographs completed in 2016 demonstrated a 49° dextroscoliosis of the thoracolumbar spine centered at T9/T10 and a 30° levoscoliosis of the lower lumbar spine centered at L4 (Fig. 1). She did not take any medications, is a nonsmoker with no comorbidities, and has a BMI of 21. The patient underwent uncomplicated posterior spinal fusion from T3 – L3 (Fig. 2). The surgery was well tolerated and the patient was deemed stable for discharge on the 6th day postoperatively.

**Fig. 3:**
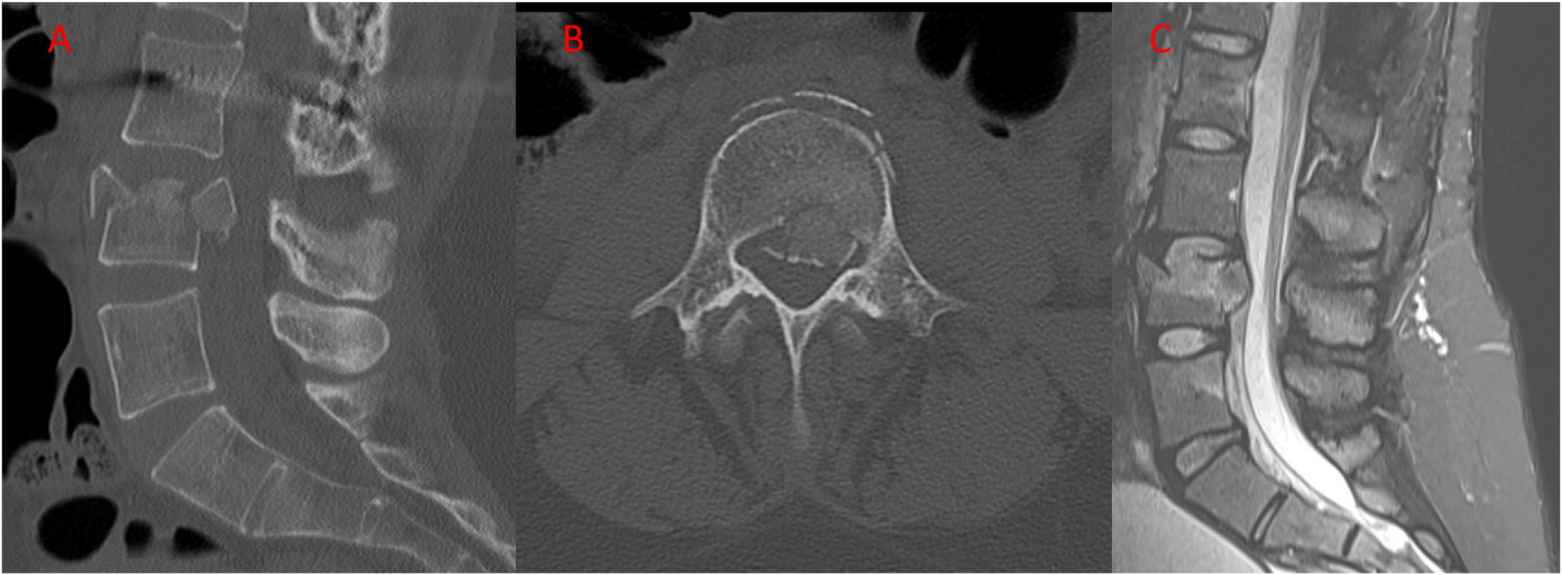
Sagittal (A) and axial (B) CT scan images showing an L4 burst fracture with central narrowing demonstrated by the sagittal MRI image (C).

Seven years postoperatively, the patient presented to the emergency department with severe low back pain that began when she fell. The patient complained of lumbar back pain exacerbated with movement and alleviated by immobilization. She denied any paresthesia or sensorimotor deficits in the extremities. Computed tomography imaging and magnetic resonance imaging demonstrated a burst fracture of the L4 vertebral body with a 9 mm of retropulsion and focal narrowing of the central canal at that level (Fig. 3). The fracture was believed to be unstable due to the magnitude of vertebral body destruction and location caudal to a long fusion- although, the posterior ligamentous complex was intact. Attempt at brace treatment was unsuccessful with inability to stand and mobilize due to unrelenting pain despite multimodal pain management.

**Fig. 4:**
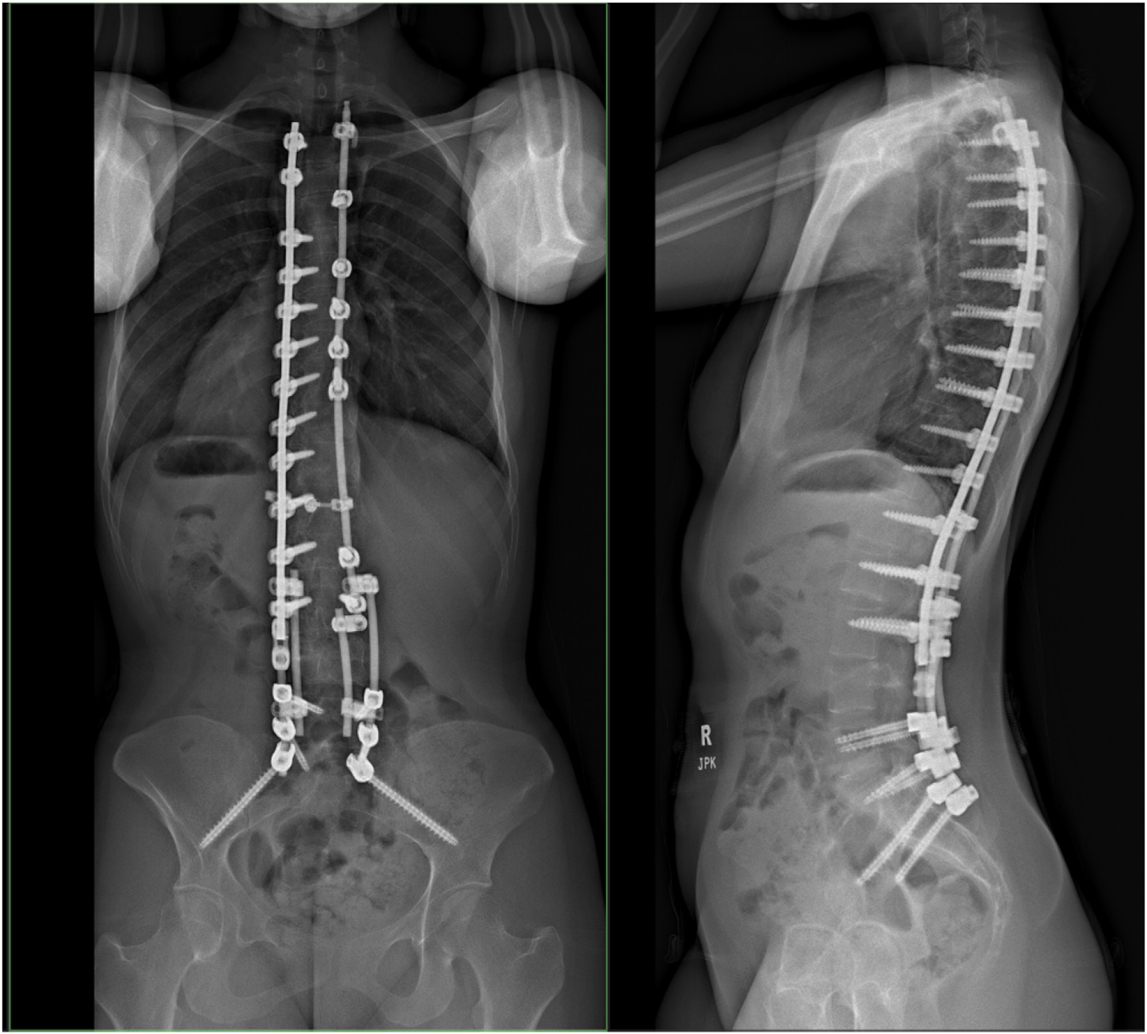
Anteroposterior and lateral plain radiograph after management of the L4 burst fracture.

The patient was taken to the operating room 2 days after the trauma for fracture fixation by posterior segmental spinal instrumentation from L2 to the ileum without fusion. The decision was made to instrument to the pelvis in order to maintain alignment in the absence of interbody support or corpectomy. There were no complications during the surgery and imaging studies upon discharge showed adequate sagittal and coronal alignment with maintenance of adequate lumbar alignment (Fig. 4).

**Fig. 5:**
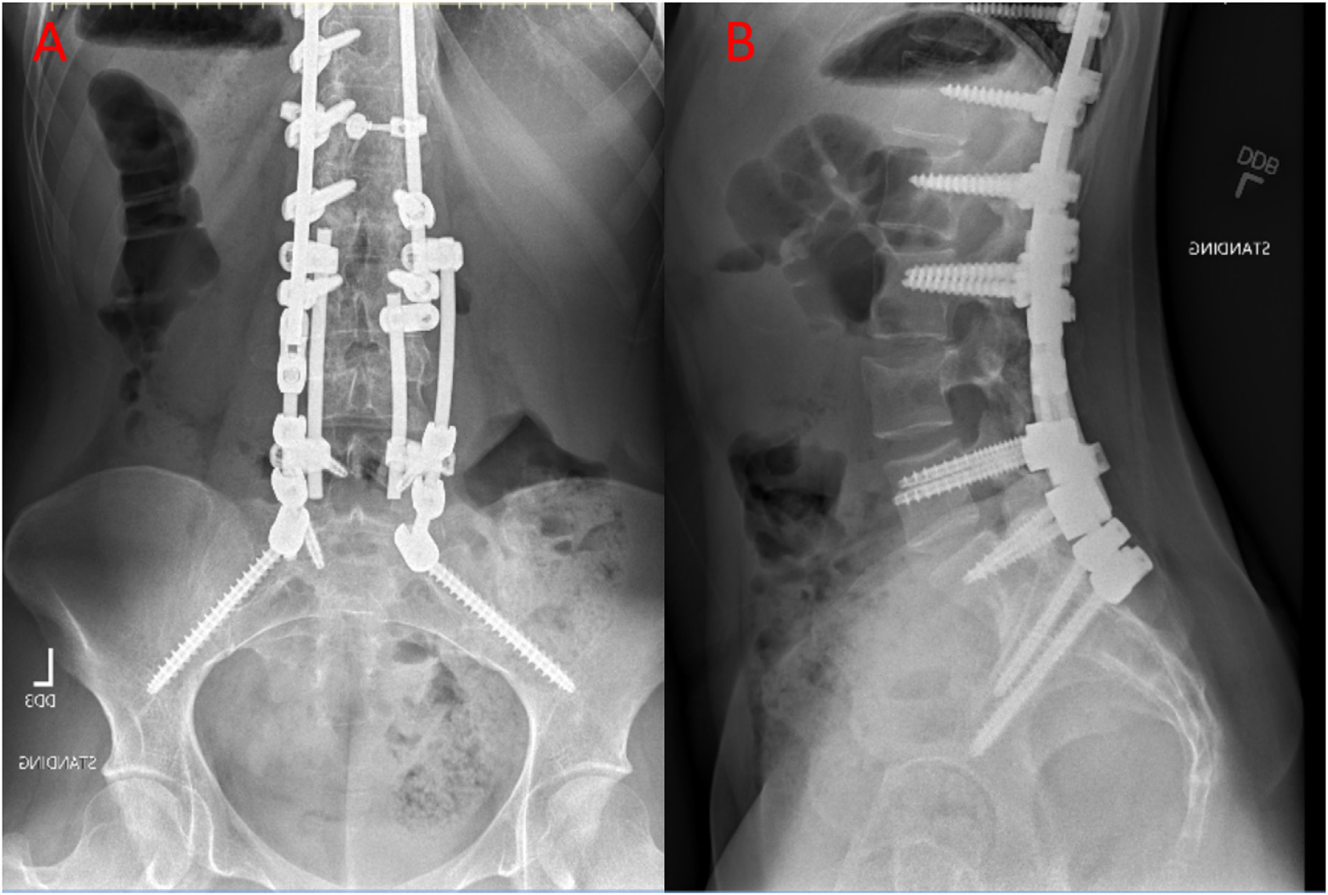
(A–B) Anteroposterior and lateral plain radiograph showing no implant related complication and a healed L4 vertebral body.

**Fig. 6:**
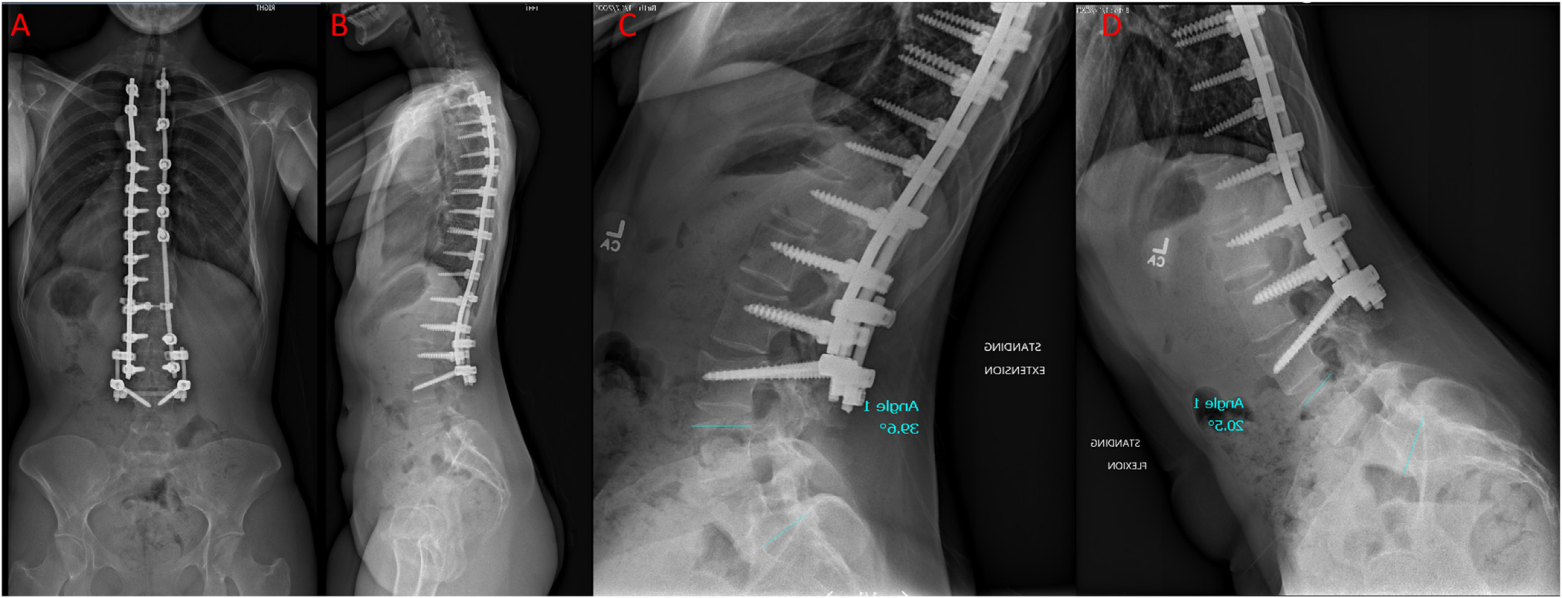
(A–B) Anteroposterior and lateral plain radiograph film after removal of the posterior instrumentation and (C–D) dynamic radiographs showing restoration of motion at L4–S1.

Fourteen months after the L4 burst fracture surgery, the patient was evaluated for continued axial lumbar spine pain and stiffness that was not relieved by medication and physical therapy. The patient desired instrumentation removal and CT showed complete healing of the fracture. Plain radiographs completed at that time demonstrated her scoliosis fixation construct with burst fracture fixation and extension to the pelvis with a fracture of L4 which appeared likely healed without any loosening or fracture of the instrumentation (Fig. 5). Therefore, the patient underwent elective removal of segmental spinal instrumentation at L5, S1, pelvis with L3–4 fusion. The screws in L5, S1, and the ileum were removed bilaterally as well as all connectors. Bilateral pedicle screws were placed in L4 and rods with connectors and setscrews were placed. The posterior elements at L3 and L4 were decorticated and allograft bone graft was placed for fusion. The decision was made to fuse to the fractured segment due to the high possibility of progressive kyphosis across the injured disc and cranial endplate of the fractured level. The patient tolerated the procedure well and was discharged on post-op day 3. On postoperative follow-up the patient reported 100% improvement in the perception of stiffness and denied any pain or complications at that time. Lateral flexion-extension lumbar radiographs demonstrated retained mobility in L4–5 and L5–S1 with 39.6° of lordosis from L4–S1 on extension which reduced to 20.5° of lordosis on flexion from L4–S1. (Fig. 6).

## Discussion

Fracture adjacent to fusion constructs are challenging to treat. Although proximal fractures are common following spinal deformity surgery, few case reports discuss post-traumatic vertebral fracture caudal to the lowest instrumented vertebra (LIV) of a previous posterior instrumentation for AIS [10], [11], [12], [13]. Of these 4 case reports, 3 implemented fusion in their constructs [11], [12], [13] while one did not mention it [10]. In this case, a posttraumatic L4 burst fracture occurred almost 7 years after a T3–L3 posterior instrumentation for AIS. The location of this injury is determined by the mechanical load distribution of the spine, and tremendous forces can be imparted on the remained unfused spine after long fusion. When a normal spine is erect, 80% to 90% of the axial compressive load is transmitted through the anterior column and the remaining force is absorbed by the posterior joints and muscles [14]. In fact, in vertebral trauma, the most commonly affected segment is the thoracolumbar junction, where the highest load transmission is present [1]. However, when the spine is instrumented at the thoracolumbar junction, where the articulation between the stiff and mobile segments exists, the load-distribution pattern is changed [11]. In our patient, due to the T3–L3 posterior instrumentation, the junction between mobile and stiff segment was pushed to L3–L4 making L4 more susceptible to a post-traumatic fracture. An additional reason could be that the instrumentation and fusion mass protected the T3–L3 region and the increased mobility between the lower and upper adjacent segments after a long spinal segment arthrodesis [10,15,16].

As for the management of thoracolumbar burst fractures, the addition of fusion with posterior instrumentation is a debated topic. For its advocators, fusion is thought to prevent fatigue and failure of the construct [4]. However, it is an additional step in surgery which has certain costs in morbidity, financial expense and permanent spinal stiffness [4]. A meta-analysis by Diniz et al.[4] comparing spinal fixation with and without fusion for thoracolumbar burst fractures showed a higher operative time and estimated blood loss in the fusion group (p < .01) with no statistically significant difference in the rate of postoperative fixation failure or kyphosis correction [4]. These findings are supported by another meta-analysis done by Lan et al.[3] adding no statistically significant difference in postoperative pain, but a higher rate of donor site-related complication in the fusion group (p < .01) [3]. Furthermore, another finding that may support nonfusion treatment in thoracolumbar burst fractures is the preservation of segmental mobility which was shown to be better in the no fusion group (p < .01) [3,4]. In addition, in 2019, the congress of neurological surgeons recommended the omission of fusion in instrumentation of thoracolumbar vertebral fractures (grade A recommendation) due to the absence of any additional benefit and its association with increased operative time and estimated blood loss [2].

## Conclusion

In this reported case, an L4 burst fracture caudal to an AIS fusion construct was treated with temporary spanning instrumentation and subsequent removal which leads to the recovery of full lower lumbar spinal mobility, and absence of any perceived postoperative stiffness or pain. Additional studies examining patients with traumatic fracture adjacent to long fusion constructs may provide further guidance on the safety of this technique. Long term follow up with also be needed to ensure ongoing mobility and satisfactory alignment of the unfused segments.

## Patient informed consent statement

Complete written informed consent was obtained from the patient for the publication of this study and accompanying images

## Declarations of Competing Interests

One or more authors declare potential competing financial interests or personal relationships as specified on required ICMJE-NASSJ Disclosure Forms
